# DGCR5 is activated by PAX5 and promotes pancreatic cancer via targeting miR-3163/TOP2A and activating Wnt/β-catenin pathway

**DOI:** 10.7150/ijbs.55636

**Published:** 2021-01-01

**Authors:** Shi-lei Liu, Chen Cai, Zi-yi Yang, Zi-you Wu, Xiang-song Wu, Xue-feng Wang, Ping Dong, Wei Gong

**Affiliations:** 1 Department of General Surgery, Xinhua Hospital, Affiliated to Shanghai Jiao Tong University School of Medicine, No. 1665 Kongjiang Road, Shanghai 200092, China; 2 Shanghai Key Laboratory of Biliary Tract Disease Research, No. 1665 Kongjiang Road, Shanghai 200092, China

**Keywords:** DGCR5, PAX5, miR-3163, TOP2A, Wnt/β-catenin pathway, pancreatic cancer

## Abstract

Long noncoding RNA DiGeorge syndrome critical region gene 5 (DGCR5) has been shown to be highly associated with cancer development. However, the biological role and molecular mechanism of DGCR5 in pancreatic cancer (PC) remains largely unknown. This study aimed to explore the role of DGCR5 in PC. It was revealed that DGCR5 was highly expressed in PC tissues compared with adjacent normal tissues and was associated with poor prognosis in PC patients. Furthermore, DGCR5 depletion inhibited the proliferation, migration and invasion by increasing apoptosis and inducing G0/G1 cell cycle arrest in vitro. Moreover, xenograft assay validated that DGCR5 promotes PC tumor growth in vivo. Mechanistically, DGCR5 was found to act as a ceRNA by sponging miR-3163 to regulate DNA topoisomerase 2-alpha (TOP2A) and inhibit Wnt/β-catenin pathway. In addition, it was found that DGCR5 downregulation could enhance the sensitivity of PC cells to gemcitabine, and ChIP assay showed that PAX5 (Paired Box 5) could bind to the promoter region of DGCR5 and increase its transcription. The results of the present study indicated that DGCR5 may be a potential diagnostic biomarker and therapeutic target for PC.

## Introduction

Pancreatic cancer (PC), one of the most rapidly lethal tumors, is the fourth most common cause of cancer mortality worldwide with the least patient survival rate of any cancer 1, 2. The primary reason for this low survival rate is the late stage at which most patients are diagnosed 1, 3. Furthermore, surgery, chemotherapy and radiation only slightly increase survival and/or relieve symptoms, but rarely cure. The 5-year survival rate is only 20% even for patients who undergo complete resection, chemotherapy and radiation 4. Therefore, novel biomarkers and therapeutic targets for PC are of extreme importance.

Data from the human whole genome and transcriptome studies revealed that more than 90% of the genome is transcribed into RNA, but less than 2% of them encodes proteins, whereas the majority of the malignant genome encodes a large number of noncoding RNAs (ncRNAs) 5-8. Long noncoding RNAs (lncRNAs), with more than 200 nucleotides in length, are a class of regulatory RNAs incapable of coding proteins 9. LncRNAs were once considered transcriptional “noise”, however, accumulating studies indicated the multi-functional role of lncRNAs in various pathological processes, in particular in the tumorigenesis 10-13. A vast number of intronic lncRNAs were found to be differentially expressed in primary and metastatic pancreatic cancer by cDNA microarrays using probes for lncRNAs 14. Various lnRNAs have been reported in PC, for example, LINC01111 was found to suppress PC aggressiveness by regulating DUSP1 expression via miR-3924^15^. Lei et al. reported that linc00976 is overexpressed in PC and promotes cancer progression via linc00976/miR-137/OTUD7B/EGFR axis 16. LncRNA DiGeorge syndrome critical region gene 5 (DGCR5) was first identified in Huntington's disease 17. A growing body of evidence shows that DGCR5 is highly associated with cancer development 18-20. However, the biological role of DGCR5 in PC remains largely unknown.

In the present study, we found that DGCR5 was overexpressed in PC tissues compared with adjacent normal tissues and its high expression was associated with poor prognosis in PC patients. Knockdown of DGCR5 inhibited the proliferation, migration and invasion by inducing cell apoptosis and G0/G1 cell cycle arrest of PC cells in vitro, and suppressed PC tumor growth in vivo. Mechanistically, DGCR5 functions as a competing endogenous RNA (ceRNA) by sponging miR-3163 to regulate TOP2A and inhibit Wnt/β-catenin pathway. Moreover, we found that DGCR5 downregulation enhanced the sensitivity of PC cells to gemcitabine, and DGCR5 was activated by the transcription factor PAX5. All these findings demonstrated that DGCR5 may serve as a potential diagnostic biomarker and therapeutic target for PC.

## Materials and Methods

### Patients and specimens

Specimens were obtained from 38 patients with a pathological diagnosis of PC who had neither chemotherapy nor radiotherapy before surgical resection, between 2015 and 2017 at the Department of General Surgery, Xinhua hospital, School of Medicine, Shanghai Jiaotong University, China. This study was approved by the Ehics Committee of Xinhua Hospital of Shanghai Jiaotong University School of Medicine.

### Cell culture

Human PC cell lines (Mia PaCa-2, PaTu8988 and PANC1), and immortalized human non-cancer pancreatic cell line (HPNE) were obtained from the Cell Bank of the Type Culture Collection of the Chinese Academy of Sciences (Shanghai, China). Cells were maintained at 37℃ in a 5% CO_2_ humidified incubator, and cultured in DMEM (Gibco) supplemented with 10% fetal bovine serum (Gibco).

### Cell transfection

DGCR5 small interfering RNAs (siRNAs), hsa-miR-3163 mimics and inhibitor, and their parental negative control (NC) were synthesized by Genomeditech (Shanghai, China) and transfected into cells using RFect reagent (Baidai, China) according to the manufacturer's protocol. The siRNA sequences are: si-DGCR5-1: 5'-GCAAUUAGCUUCAGCUCUA-3', si-DGCR5-2: 5'-GCGAGAUGUUAUUUCUGAA-3', si-DGCR5-3: 5'-GGUGGUGCACGAGAGUCUA-3'; si-TOP2A: 5′-CUAUUCAUCUUUCCCGAGA-3′; si-PAX5-1: 5'-GAGGAUAGUGGAACUUGCUCAUCAA-3', si-PAX5-2: 5'-GGT​AAT​TGG​AGG​ATC​CAA​A-3'. Lentiviruses expressing full-length of DGCR5, sh-DGCR5 (5'-GCAAUUAGCUUCAGCUCUAdTdT-3') and NC were constructed by Genomeditech (Shanghai, China). Cells were infected at a multiplicity of infection (MOI) of 90 in complete culture medium for 48 hours, and then subjected to selection with 1μg/ml puromycin for 1 week to construct stable-transfected cells. Transfection efficiency was verified by quantitative real-time PCR (qRT-PCR).

### Quantitative real-time PCR

Total RNA was isolated by TRIzol reagent according to the manufacturer's instructions. The Mir-X miRNA qRT-PCR TB Green Kit (Takara) was used for converting miR-3163, other cDNAs were generated by PrimScript Reverse Transcriptase (Takara) according to the manufacturer's instruction. QRT-PCR was performed using SYBR Premix Ex Taq II (Takara) on StepOnePlusTM Real-time PCR system (Applied Biosystems, USA). 2^-ΔΔCT^ method was used for the quantification of gene expression. The primer sequences were synthesized by HuaGene Biotech (Shanghai, China) and listed as followed: DGCR5 forward: 5ʹ-ATTTTCCCAGTCTGGCGGAG-3ʹ, reverse: 5ʹ-AGGGCCCCATTATGACTCCT‐3ʹ; TOP2A forward: 5ʹ-ACCATTGCAGCCTGTAAATGA-3ʹ, reverse: 5ʹ-GGGCGGAGCAAAATATGTTCC‐3ʹ; GAPDH forward: 5′-CAACAGCCTCAAGATCATCAGC-3′, reverse: 5′-TTCTAGACGGCAGGTCAGGTC-3′; PAX5 forward: 5'-ACTTGCTCATCAAGGTGTCAG-3'; reverse: 5'-TCCTCCAATTACCCCAGGCTT-3'; U6 forward: 5'-AAAGCAAATCATCGGACGACC-3', reverse: 5'-GTACAACACATTGTTTCCTCGGA-3'.

### Fluorescence in Situ Hybridization (FISH) assay

Cellular fractionation of DGCR5 was measured using DGCR5 FISH probes and kit designed and synthesized by GenePharma (Shanghai, China) following the manufacturer's instructions.

### CCK-8 assay

Cells were plated into a 96-well plates overnight at the density of 2000 cells/well with complete culture medium DMEM. Each well was replaced with 10 μl CCK-8 and 90 μl complete medium. After incubating for 2 hours at 37℃ away from light, optical density (OD) value at 450nm was detected through a microplate reader (Bio-Tek).

### Colony formation assay

500 treated cells were plated in each well of a 6-well plate for 7 to 10 days. The cells were fixed with 4% paraformaldehyde and stained with 0.1% crystal violet. After washing and drying the plate, cell colonies were counted and analyzed.

### 5‐Ethynyl‐2′‐deoxyuridine (EdU)-488 proliferation assay

BeyoClickTM EdU-488 proliferation assay (Beyotime, Shanghai, China) was used to examine the effect of DGCR5 on cell proliferation according to the manufacturer's protocol. Cells were incubated with 10μM EdU for 2 hours, stained with Azide488 and Hoechst 33342 then visualized under a fluorescence microscope (Leica).

### Transwell assay

Cell migration and invasion capabilities were measured using Transwell chamber inserts (Corning, NY, USA) and Corning BioCoat Growth Factor Reduced Matrigel Invasion Chambers (Corning, NY, USA), respectively. 750 μl DMEM with 10% FBS was added into the lower chamber, 3 × 10^4^ cells with 200 μl serum-free medium were plated in the upper chamber. After incubation for 24 hours, cells were fixed with 4% paraformaldehyde and stained with 0.1% crystal violet. Cells remaining on the upper membrane were gently washed with PBS and scraped by cotton swabs. Stained cells on the bottom of the chamber were counted under a microscope on 5 random fields.

### Analysis of cell apoptosis and cell cycle by flow cytometry

Cells in apoptosis were detected using an Annexin V/PI apoptosis assay kit (Beyotime, Shanghai, China). In brief, the transfected cells were collected and re-suspended with PBS, stained with 5 μl Annexin V-FITC and/or 5 μl PI for 30 minutes at room temperature shielded from light. Cell apoptosis was immediately determined by flow cytometry.

For cell cycle analysis, transfected cells were harvested and fixed with 75% ethanol at 4℃ overnight. After fixation, cells were washed and re-suspended with PBS and incubated with 10mg/ml RNase and 1mg/ml PI at 37 ℃ for 30 minutes in the dark. The cell cycle was analyzed by flow cytometry.

### Western blot analysis

Protein was extracted from transfected cells with RIPA lysis buffer. Equal amounts of protein samples were loaded and separated by SDS-PAGE and transferred onto PVDF membranes. The membranes were blocked with 5% skim milk in TBST for 1 hour at room temperature. Then, membranes were incubated with diluted primary antibodies at 4℃ overnight. The antibody against PAX5 was obtained from Abcam, other antibodies were acquired from Cell Signaling Technology. Next, membranes were rinsed with TBST 3 times, followed by incubating with secondary antibody for 1 hour, rinsed again with TBST and finally determined by a Gel Doc 2000 (Bio-Rad).

### Xenograft model

Nude mice (female, 4 weeks of age) with 18-22g were obtained from the Shanghai Laboratory Animal Center of the Chinese Academy of Sciences (Shanghai, China). In this assay, nude mice were randomly divided into 4 groups (n = 5 per group). PaTu8988 cells (1 × 10^7^ in 200 μL PBS) transfected with LV-NC (2 groups), LV-shDGCR5 or LV-DGCR5 were injected hypodermically into the left axilla of the mice. Starting from Day 7, tumor volumes were estimated every 7 days by measuring the tumor length and width by caliper. Tumor volume was calculated as π/6 × width^2^ × length. All mice were sacrificed by dislocation after 4 weeks, tumors were collected for further assays. The study was approved by the Ethics Committee of Xinhua Hospital Affiliated to Shanghai Jiaotong University School of Medicine.

### Immunohistochemistry

Immunohistochemistry was performed according to the standard procedure. The antibodies against Ki-67, PCNA, N-cadherin, TOP2A and β-Catenin were obtained from Cell Signaling Technology. Each slice was observed with 5 random fields under a microscope (Leica), and the level of expression was assessed by Image J software.

### RNA sequencing

Total RNA from NC or DGCR5-knockdown Mia PaCa-2 and PaTu8988 cells was extracted and subjected to mRNA sequencing on the BGISEQ-500 platform at the Beijing Genomics Institute (BGI, Shenzhen, China). The differentially expressed genes in both cell lines were selected that met the criteria of a fold change ≤ -1.0 or ≥ 0.85 and P ≤ 0.05 after DGCR5 knockdown. The analysis were performed using the BGI online analysis system.

### Bioinformatics analysis

The DIANA-LncBase (http://carolina.imis.athena-innovation.gr) and TargetScan (http://www.targetscan.org) databases were used to search the potential miRNA that interacts between DGCR5 and TOP2A. The transcription factor PAX and its binding sites in the promoter region of DGCR5 was identified by PROMO (http://alggen.lsi.upc.es/cgi-bin/promo_v3/promo/promoinit.cgi?dirDB=TF_8.3), GeneCards (http://genecards.org) and JASPAR (http://jaspar.genereg.net/).

### Dual-luciferase reporter assays

293T cells were plated at 2 × 10^4^ cells/well in 24-well plates overnight. Then the cells were cotransfected with WT-DGCR5, MUT-DGCR5, WT-TOP2A, MUT-TOP2A reporter plasmids (Genomeditech, Shanghai), miR-3163 mimics, NC mimics, miR-3163 inhibitor or NC inhibitor. After 48 hours of incubation, the relative luciferase activities were determined by Dual-Luciferase Reporter Assay System (Promega) according to the manufacturer's protocol.

### ChIP assay

ChIP assays were performed with Mia PaCa-2 and PaTu8988 cells via ChIP Assay Kit (Beyotime, Shanghai, China) following the manufacturer's instruction. Anti-PAX5 antibody and IgG antibody were obtained from Abcam. The ChIP samples were analyzed via qRT-PCR and the PCR products were verified by 2% agarose gel electrophoresis. The primer sequences of PAX5-DGCR5 are as follow: forward: 5'-TGCTTGTTTAGGGGTCAGGC-3'; reverse: 5'-CTGAGGTGGCTCAACAGGTC-3'.

### Statistical analysis

Statistical analyses were performed using GraphPad Prism 7 and the results were presented as mean ± standard deviations (SD). The student's t tests were performed for comparisons between 2 groups and one-way ANOVA was used for multiple group comparisons. The Chi-square test was conducted to compare clinicopathological features of the patients with DGCR5 expression. The Kaplan-Meier plots and log-rank tests were used for the overall survival analysis. The multivariate cox proportional hazards analysis was applied for the multivariate survival analysis. The correlation between DGCR5 and TOP2A was analyzed by Spearman's correlation test. Data were presented as mean ± SD of at least three independent experiments. Results with P < 0.05 were considered statistical significant.

## Results

### DGCR5 is upregulated in PC tissues and cell lines

**Figure 1:**
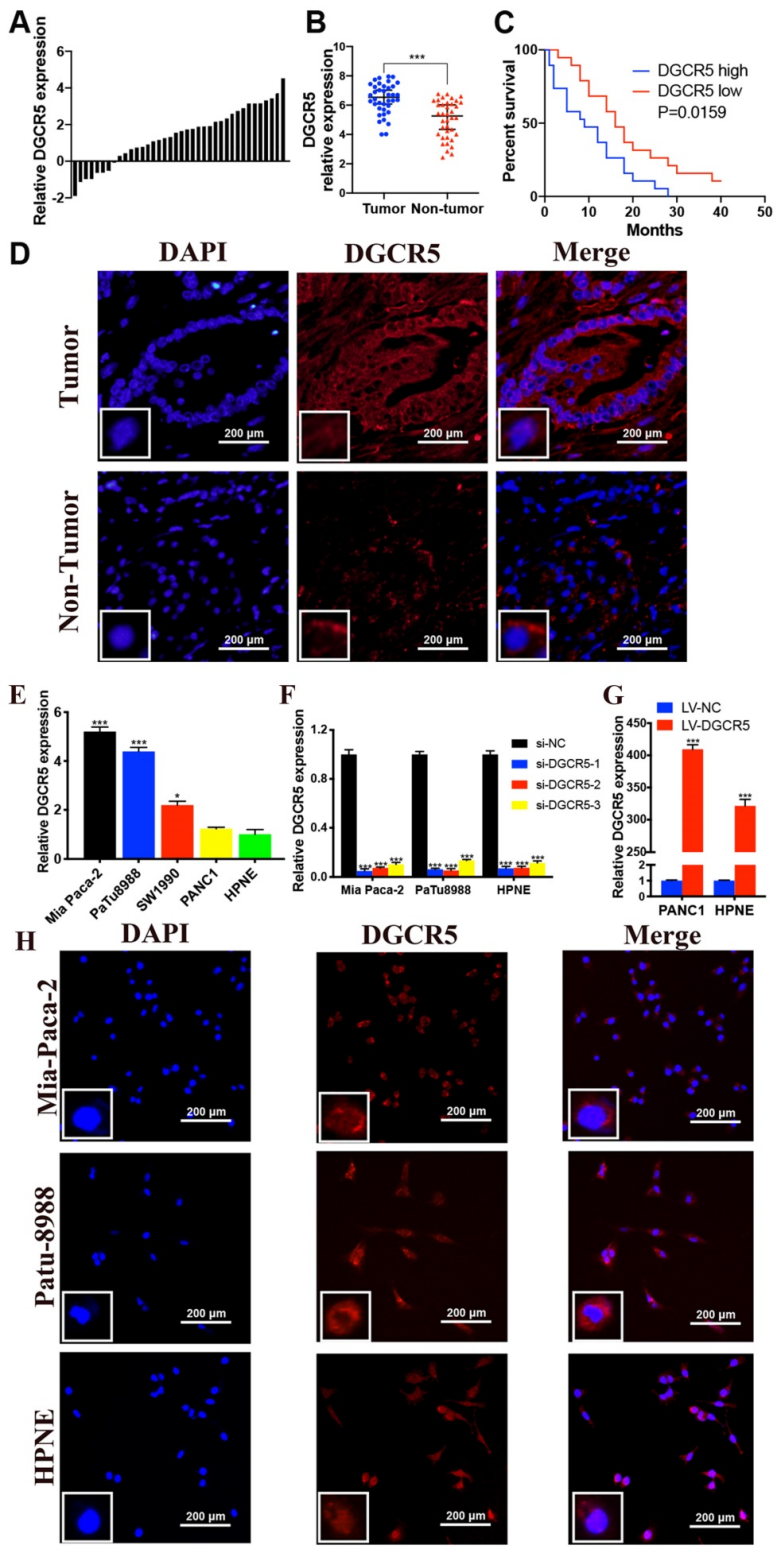
** High expression of DGCR5 is observed in PC tissues and cell lines.** (A) DGCR5 expression was investigated in PC tissue compared with its adjacent normal tissues. (B) The relative DGCR5 expression in PC tissues and adjacent normal tissues. (C) The correlation of DGCR5 expression with overall survival rate of PC patients. (D) FISH assays were performed to detect the subcellular location of DGCR5 in patient samples. (E) The relative DGCR5 expression was measured in cell lines by qRT-PCR. (F) DGCR5 expression level was examined using qRT-PCR in cell lines after transfected with siRNAs. (G) The DGCR5 expression level in cell lines after viral infection. (H) Analysis of the intracellular distribution of DGCR5 in cell lines by FISH. s* P < 0.05, ** P < 0.01, *** P < 0.001.

To determine the biological role of DGCR5 in PC, we measured its expression in 38 pairs of PC tissues and adjacent non-cancer tissues by qRT-PCR. The results indicated that DGCR5 expression was notably incresed in PC tissues compare with non-tumor tissues (Figure 1A and B). Using the median expression level of DGCR5 as a cutoff, patients with high DGCR5 expression had obviously poorer overall survival (OS) than those with low DGCR5 expression (P=0.0159, Figure 1C). Moreover, we investigated the association between DGCR5 expression and clinicopathologic parameters in those PC patients. As shown in Table 1, DGCR5 expression was positively associated with histological grade (P<0.0001), TNM stage (P=0.0436), lymphatic invasion (P=0.001) and distant metastasis (P=0.0164). However, there was no significant correlation between DGCR5 expression and gender, age, tumor size or vascular infiltration. The multivariate survival analysis showed that DGCR5 expression was an independent risk factor that negatively associated with prognosis of PC patients (Table 2, *p*< 0.05). As expected, DGCR5 was overexpressed in PC cell lines (Mia PaCa-2, PuTu8988 and PANC1) compared with normal human pancreatic cell (HPNE) (Figure 1E). Mia PaCa-2 and PuTu8988 cell lines were selected to further explore the effect of specific depletion of DGCR5, while PANC1 cell line was selected to investigate the effect of overexpression of DGCR5 in PC (Figure 1F and G). RNA Fluorescence in situ hybridization (FISH) analysis in PC tumor tissues and cell lines displayed that DGCR5 was primarily located in the cytoplasm (Figure 1D and H).

### DGCR5 promotes PC cell proliferation in vitro

**Figure 2:**
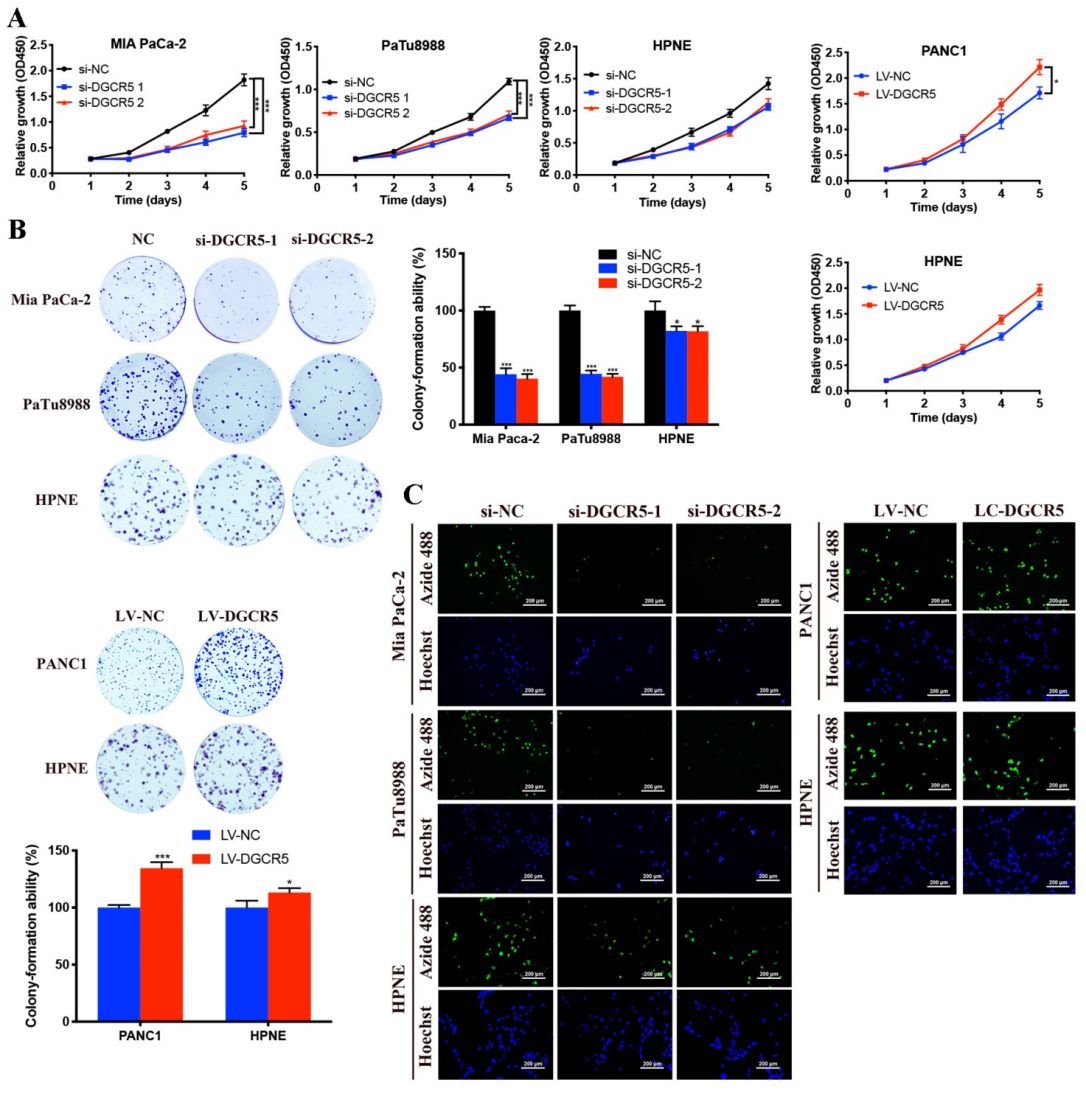
** DGCR5 promotes PC cell proliferation in vitro.** (A) Cell viability was detected by CCK-8 assays in cell lines after DGCR5 knockdown or overexpression. (B) Colony formation was applied to measure the proliferation of cell lines after treatment with siRNAs or lentivirus. (C) Proliferation of cells after DGCR5 silencing or enhancing was determined by detecting DNA synthesis through EdU assay. * P < 0.05, ** P < 0.01, *** P < 0.001.

To explore the effects of DGCR5 on PC cell proliferation, CCK-8, colony formation and EdU assays were carried out in our study. As shown in Figure 2A, the cell growth of Mia PaCa-2 and PaTu8988 cells was downregulated after DGCR5 knockdown, while DGCR5 overexpression had the opposite effects in PANC1 cells. In addition, the effect of DGCR5 on HPNE was milder compared with that on PC cell lines, though the results had the same change trend. Consistent with the CCK-8 results, the colony formation and EdU assays demonstrated that downregulation of DGCR5 significantly suppressed the ability of colony formation and proliferation in PC cells, in contrast, overexpression of DGCR5 enhanced those abilities (Figure 2B and C).

### Knockdown of DGCR5 induces apoptosis and G0/G1 cell cycle arrest in PC cells

**Figure 3:**
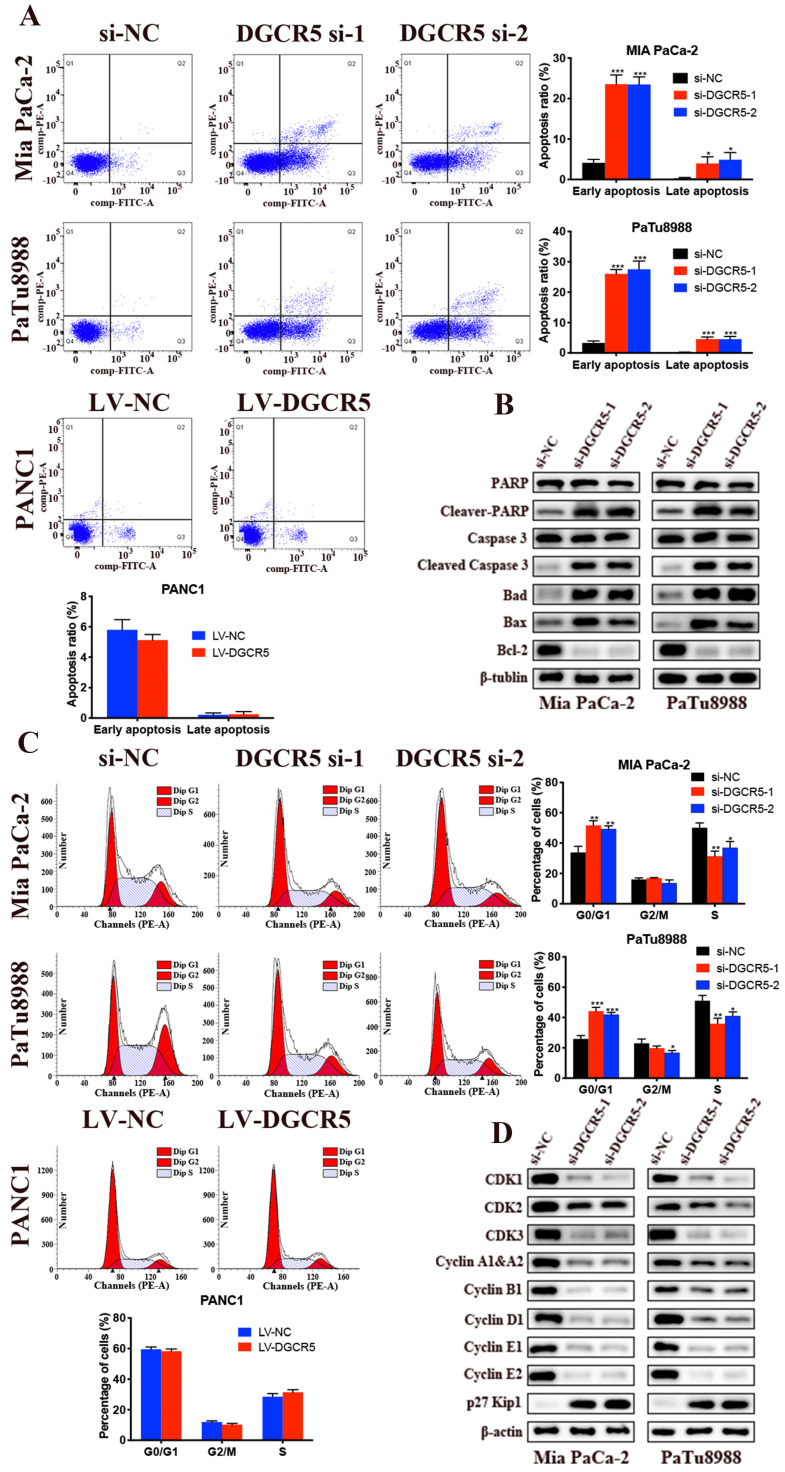
** Knockdown of DGCR5 induces cell apoptosis and G0/G1 phase arrest in PC cells.** (A) Cell apoptosis analysis of Mia PaCa-2, PaTu8988 and PANC1 cells was measured by flow cytometry. (B) The expression of cell apoptosis-related proteins in DGCR5-knowckdown PC cells was detected by western blot. (C) Phase of cell cycle analysis based on flow cytometry showed that PC cells were blocked in G0/G1 phase after DGCR5 silencing. (D) The protein level of key cell cycle regulators in DGCR5-knockdown PC cells was examined with western blot method. * P < 0.05, ** P < 0.01, *** P < 0.001.

We examined the effects of DGCR5 on cell apoptosis through flow cytometry. The results showed that both the early and late stages of apoptosis after DGCR5 knockdown was remarkably higher than that in NC group (Figure 3A). Moreover, downregulation of DGCR5 led to a significant increase in G0/G1 phase population (Figure 3C). To further characterize the mechanism of apoptosis and cell cycle arrest by DGCR5 knockdown, we examined the expression levels of the key cell apoptosis and cell cycle regulators by western blot. As shown in Figure 3B and D, DGCR5 knockdown markedly increased the protein levels of cleaved-PARP, cleaved-caspase 3, bad, bax, p27 Kip1and reduced the levels of Bcl-2, CDK1, CDK2, CDK3, cyclin A1&A2, B1, D1,E1,E2. Collectively, these data revealed that DGCR5 plays a critical role in the regulation of PC cell apoptosis and cell cycle.

### DGCR5 enhances migration, invasion and EMT process in PC cells

**Figure 4:**
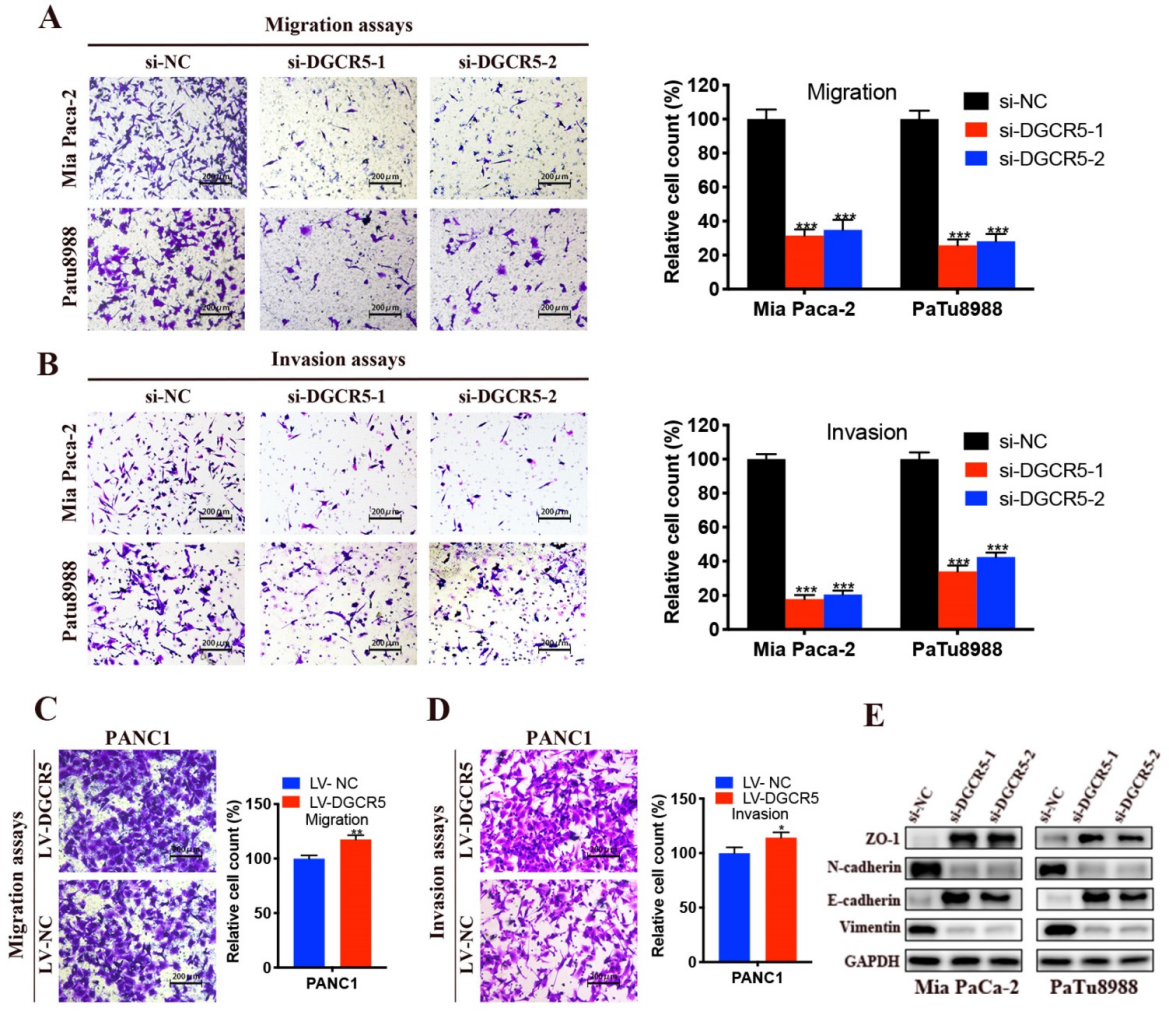
** DGCR5 promotes PC cell migration, invasion and EMT process.** (A - D) Migration assays (A and C) and invasion assays (B and D) were performed in DGCR5-silencing Mia PaCa-2 and PaTu8988 cells and DGCR5-overexpressing PANC1 cells. (E) Western blot analysis of ZO-1, N-cadherin, E-cadherin and vimentin. * P < 0.05, ** P < 0.01, *** P < 0.001.

Given that DGCR5 expression was positively related to the distant metastasis in PC patients, we reasoned that DGCR5 may control the migration and invasion of PC cells. To investigate this, we performed transwell migration and invasion assays. As shown in Figure 4A-D, cell migration and invasion ability was remarkably reduced after DGCR5 knockdown, conversely, enhanced after DGCR5 overexpression. It is well established that epithelial-mesenchymal transition (EMT) is a vital step for the promotion of tumor metastasis^21^, thus we examined pivotal EMT markers by western blot. It was found that DGCR5 knockdown increased the expression of ZO-1, E-cadherin while decreased the expression of N-cadherin and vimentin (Figure 4E). All these results suggested that DGCR5 stimulated PC cells to undergo EMT and metastasis.

### DGCR5 promotes PC tumor growth in vivo

**Figure 5:**
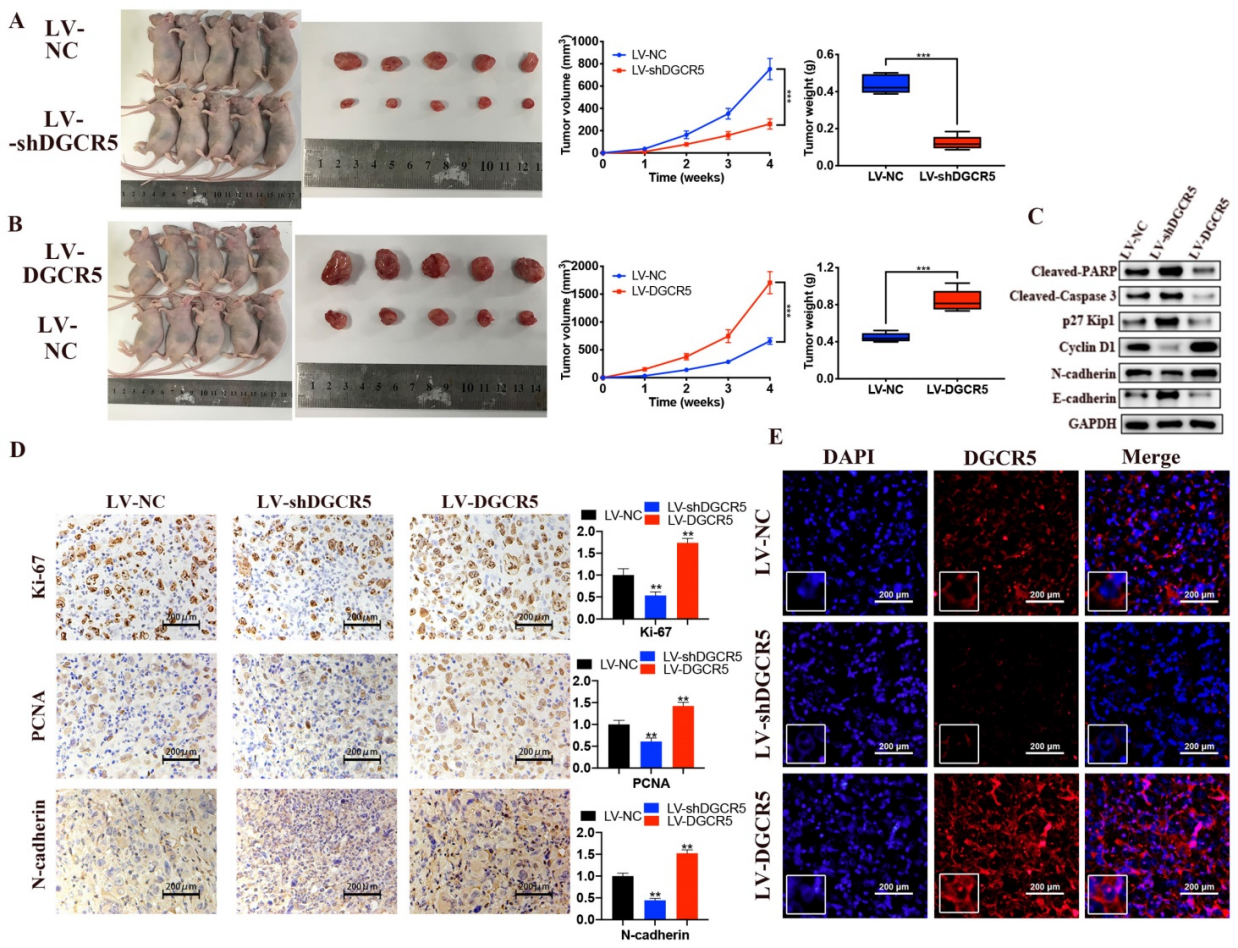
** DGCR5 enhances the growth of PC cell in vivo.** (A and B) DGCR5 knockdown suppressed while DGCR5 overexpression promoted the growth of PC cells in vivo. (C) Western blot analysis of cleaved-PARP, -caspase 3, p27 Kip1, cyclin D1, N-cadherin and E-cadherin. (D) The expression of Ki-67, PCNA and N-cadherin by IHC staining. (E) FISH assay for DGCR5 expression in xenograft model tissues. * P < 0.05, ** P < 0.01, *** P < 0.001.

To further demonstrate the role of DGCR5 in PC, animal study was carried out. Compared with the NC group, the tumor volume and weight was significantly decreased in LV-shDGCR5 group while increased in LV-DGCR5 group (Figure 5A and B). The western blot results showed that DGCR5 knockdown increased the expression of cleaved-PARP, cleaved-caspase 3, p27 Kip1, E-cadherin while decreased the expression of cyclin D1 and N-cadherin (Figure 5C). In addition, IHC staining showed that ki-67, PCNA and N-cadherin expression was upregulated in LV-shDGCR5 group and downregulated in LV-DGCR5 group (Figure 5D). RNA FISH assay using xenograft tumor tissues further demonstrated that DGCR5 mainly located in the cytoplasm (Figure 5E). The above results revealed the tumor-promoting role of DGCR5 in PC.

### DGCR5 promotes PC progression via miR-3163/TOP2A/Wnt/β-catenin pathway

**Figure 6:**
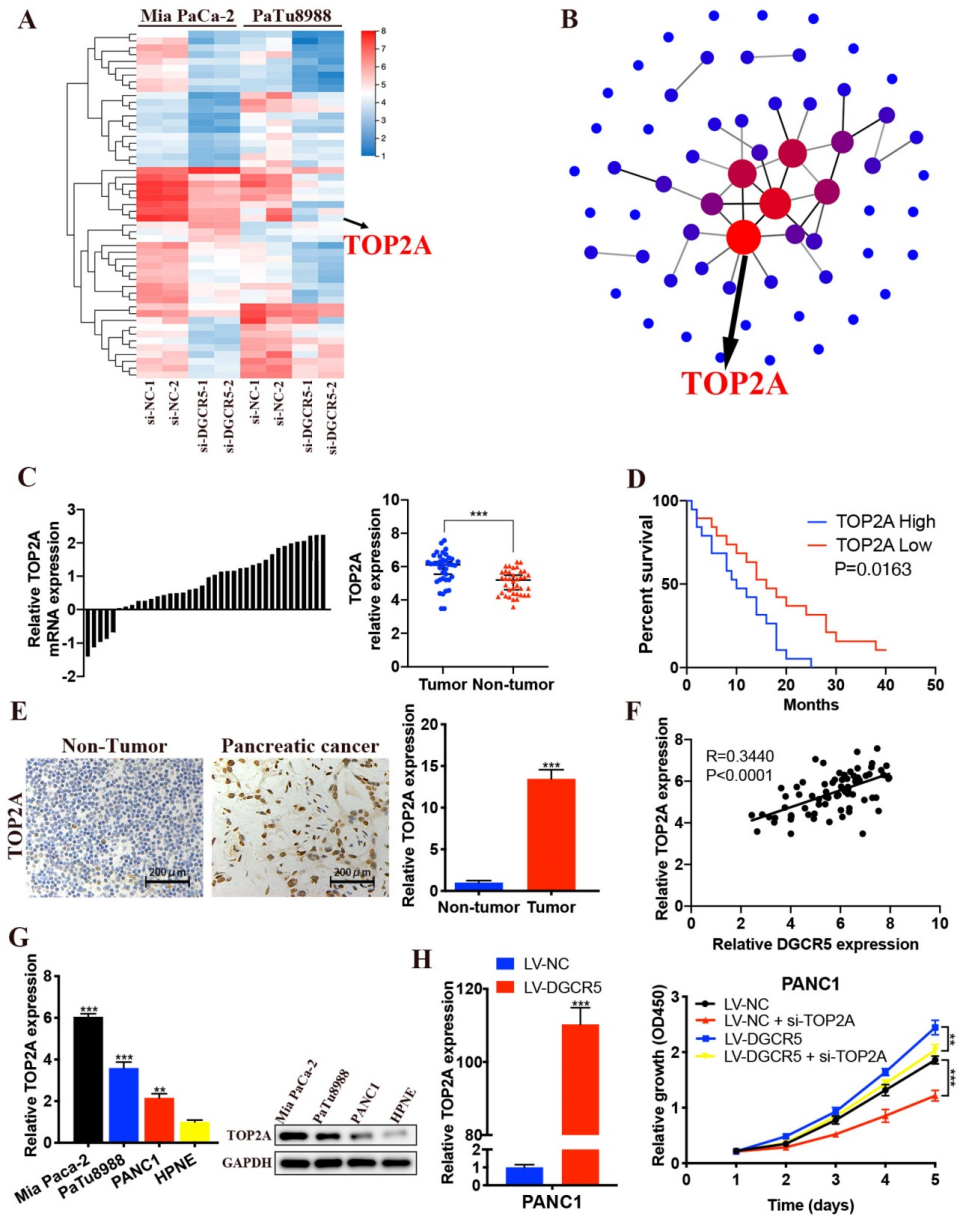
** DGCR5 increases PC cell proliferation by regulating TOP2A.** (A) Heatmap of 61 most significantly differentially expressed genes (DEGs) expression in Mia PaCa-2 and PaTu8988 cells after DGCR5 knockdown by RNA-sequencing assay. (B) Protein-protein interaction (PPI) networks of the DEGs. (C) Relative expression of TOP2A in 38 paired PC samples. (D) Kaplan-Meier survival curves of 38 PC patients based on TOP2A expression. (E) TOP2A expression detected by IHC assay in PC and adjacent non-tumor tissue. (F) Pearson correlation analysis showed that TOP2A was positively correlated with DGCR5. (G) TOP2A was overexpressed in PC cell lines. (H) PANC1 cell proliferation curve using CCK-8 assay after DGCR5 overexpression and/or TOP2A knockdown. * P < 0.05, ** P < 0.01, *** P < 0.001.

We performed RNA sequencing to further explore the underlying mechanism of DGCR5 in PC progression. The 61 most significantly differentially expressed genes (DEGs) in Mia PaCa-2 and PaTu8988 cells after DGCR5 knockdown were selected, including 49 downregulated genes and 12 upregulated genes (Figure 6A). Based on the PPI network of DEGs, TOP2A was identified for being the core of the PPI network (Figure 6B). Moreover, it has been reported that TOP2A could induce malignant character of PC progression 22. Therefore, we assumed that TOP2A was a downstream gene of DGCR5 and played an important role in the oncogenic effects of DGCR5. Then we investigate the association between the expression of TOP2A and PC. As shown in Figure 6C-E, increased expression of TOP2A was found in PC tissues and was associated with shorter overall survival in PC patients. Moreover, there was a positive association between the expression of TOP2A and DGCR5 in PC tissues (Figure 6F). We detected the expression level of TOP2A in PC cell lines (Mia PaCa-2, PaTu8988 and PANC1) and HPNE by qRT-PCR and western blot. The results showed that TOP2A was significantly overexpressed in PC cells than normal pancreatic cell (Figure 6G).

In addition, we found that DGCR5 overexpression increased the expression of TOP2A, and TOP2A knockdown inhibited the cell proliferation that promoted by DGCR5 upregulation (Figure 6H).

**Figure 7:**
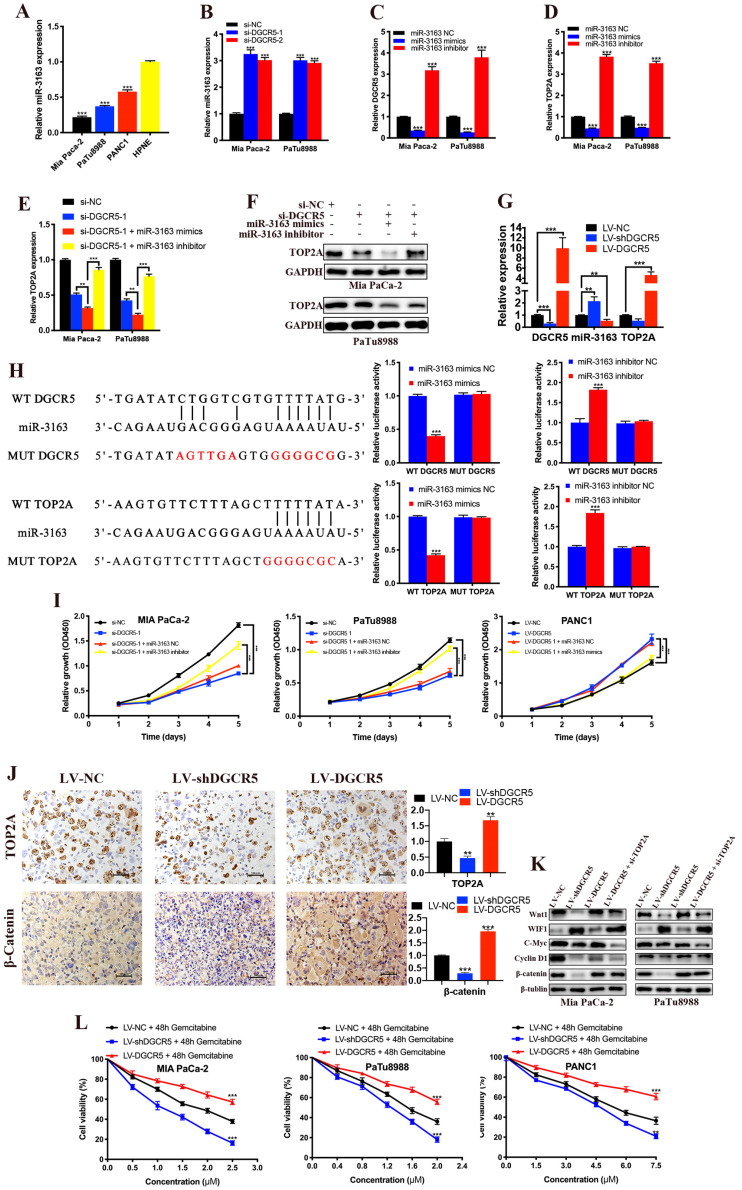
** DGCR5 promotes PC by sponging miR-3163 to target TOP2A and activating Wnt/β-catenin pathway.** (A) MiR-3163 expression was decreased in PC cell lines. (B) DGCR5 knockdown enhanced the expression of miR-3163. (C and D) Expression of DGCR5 and TOP2A in Mia PaCa-2 and PaTu8988 cells after miR-3163 mimics or inhibitor transfection. (E and F) The mRNA and protein expression level of TOP2A in DGCR5-silencing PC cells with inhibition or overexpression of miR-3163. (G) QRT-PCR was performed to detect the expression of DGCR5, miR-3163 and TOP2A in PC xenografts. (H) The direct binding between DGCR5 or TOP2A and miR-3163 was verified by dual-luciferase reporter assay. (I) Cell proliferation assays for Mia PaCa-2 and PaTu8988 cells co-transfected with si-DGCR5 and/or miR-3163 inhibitor, and DGCR5-overexpressing PANC1 cells transfected with miR-3163 mimics. (J) The expression of TOP2A and β-catenin in xenograft model tissues was measured by IHC assay. (K) Expression of Wnt1, WIF1, C-Myc, Cyclin D1 in Mia PaCa-2 and PaTu8988 cells after DGCR5 knockdown or overexpression and/or TOP2A knockdown. (L) The viability of DGCR5-depleted and enhanced PC cells after 48 hours gemcitabine treatment was determined by CCK-8 assay. * P < 0.05, ** P < 0.01, *** P < 0.001.

It is widely known that lncRNAs can act as ceRNAs to regulate mRNA in the cytoplasm^23^, ^24^, and we have previously demonstrated that DGCR5 primarily located in the cytoplasm of PC cell. Thus, we used DIANA-LncBase and TargetScan databases to search for miRNAs that might be the bridge between DGCR5 and TOP2A. MiR-3163 was screened out from the predicted targets for it had the most potential to interact with both DGCR5 and TOP2A, and has been reported involved in the cross-talk between lncRNA and cancer^25^, ^26^. As shown in Figure 7A-D, miR-3163 expression was inversely related to the expression of DGCR5 and TOP2A. What's more, the expression of TOP2A increased by DGCR5 knockdown was further increased by miR-3163 overexpression while abolished by miR-3163 inhibition (Figure 7E and F). The expression of DGCR5, miR-3163 and TOP2A in PC xenografts was examined by qRT-PCR (Figure 7G). Then we investigated whether miR-3163 directly bind to DGCR5 and TOP2A. The results of dual-luciferase reporter assay indicated that luciferase activity of WT-DGCR5 and WT-TOP2A reporter was significantly reduced by miR-3163 mimics while increased by miR-3163 inhibitor, moreover, these changes were not occurred in the MUT-DGCR5 and MUT-TOP2A reporter (Figure 7H). Next, we conducted CCK-8 assays to explore whether DGCR5 promotes the PC cell proliferation via miR-3163. As shown in Figure 7I, miR-3163 inhibitor and mimics significantly reduced the proliferation effects of DGCR5 knockdown and overexpression, respectively. To sum up, the above results demonstrated that DGCR5 promotes PC proliferation as a ceRNA to target TOP2A by sponging miR-3163.

As we know, the Wnt/β-catenin signaling pathway is particularly relevant in PC and it has been reported that TOP2A promoted malignant progresssion of PC through activating this pathway^22^, ^27^. Hence, we explored whether DGCR5 promotes PC via regulating TOP2A and wnt/β-catenin pathway. IHC results showed that the expression of TOP2A and β-catenin was reduced by DGCR5 knockdown while increased by DGCR5 overexpression in vivo (Figure 7J). As shown in Figure 7K, DGCR5 downregulation significantly suppressed the expression of Wnt/β-catenin pathway-related proteins. Conversely, DGCR5 upregulation resulted in an increase in the expression of these proteins, while these changes were reversed by TOP2A downregulation. Then we undertook rescue experiments to further confirm whether Wnt/β-catenin pathway was involved. PANC1 cells were transfected with LV-NC or LV-DGCR5, and then treated with 0.1% DMSO or 5μM Wnt/β-catenin pathway inhibitor MASB (methyl 3-{[(4-methylphenyl)sulfonyl]amino}benzoate, MedChemExpress). The results of CCK-8, clone formation and transwell assays indicated that MASB could rescue the cell proliferation, migration and invasion that enhanced by DGCR5 (Figure S1).

In summary, the above data verified that DGCR5 regulates the PC progression by sponging miR-3163 to target TOP2A and activating Wnt/β-catenin pathway.

### DGCR5 induces gemcitabine resistance of PC cells

Our previous data has proved TOP2A as a downstream gene of DGCR5, which was found associated with gemcitabine resistance in PC patients by gene expression analysis^28^. CCK-8 assays showed that gemcitabine repressed PC cell viability in a dose-dependent manner, and as expected, the expression levels of DGCR5 was negatively associated with gemcitabine sensitivity in PC cells (Figure 7L), suggesting that inhibition of DGCR5 may be a novel strategy for anti-gemcitabine resistance in PC.

### PAX5 directly promotes transcription of DGCR5 in PC cells

**Figure 8:**
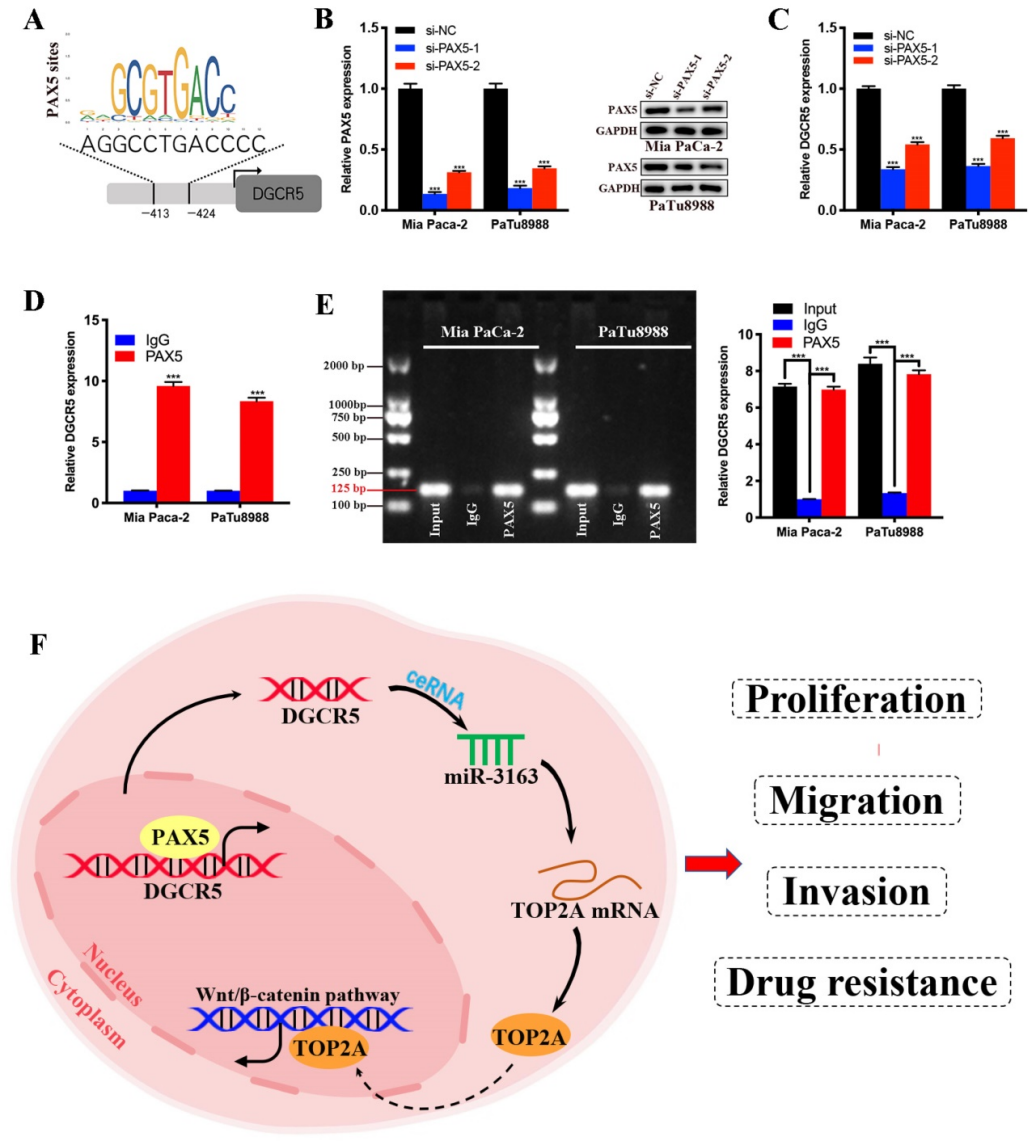
** PAX5 activates DGCR5 in PC cells.** (A) Binding motif of PAX5 and the predicted binding sites of PAX5 within the DGCR5 promoter region provided by JASPAR. (B) QRT-PCR and western blot were used to determine the knockdown efficiency of PAX5 in Mia PaCa-2 and PaTu8988 cells. (C) PAX5 knockdown decreased the DGCR5 expression in PC cells. (D) and (E) qRT-PCR and agarose gel electrophoresis of the ChIP products confirmed the direct binding between PAX5 and DGCR5 promoter. (F) The mechanism of DGCR5 regulatory network in PC. DGCR5 was enhanced by PAX5 and acted as a ceRNA for miR-3163 to regulate TOP2A and activate Wnt/β-catenin pathway, thus resulting in promoted cell proliferation, metastasis and drug resistance in PC. * P < 0.05, ** P < 0.01, *** P < 0.001.

To further investigate the transcriptional regulatory mechanism of DGCR5 in PC, we search for the transcription factors that may regulate DGCR5 by performing bioinformatics analysis using PROMO, GeneCards and JASPAR databases. PAX5 was identified from the candidate transcription factors for its high predicted scores in all databases, and the predicted binding sites of PAX5 in the promoter sequence of DGCR5 are shown in Figure 8A. To confirm this hypothesis, we first performed qRT-PCR and western blot assays, and found that PAX5 knockdown resulted in a decrease in DGCR5 expression in PC cells (Figure 8B and C). Next, we conducted ChIP assays in Mia PaCa-2 and PaTu8988 cells, followed by qRT-PCR and agarose gel electrophoresis. The results showed that the presence of the DGCR5 gene promoter region was specifically enriched by PAX5 antibody but not IgG antibody (Figure 8D and E). These data suggest that PAX5 acts as a transcriptional activator of DGCR5.

## Discussion

There is increasing evidences that lncRNAs exert important roles and to act through a variety of molecular mechanisms, such as ceRNAs, in the development and progression of various cancers, including PC 15, 16, 29. LncRNA DGCR5 has not been extensively reported in cancers since it was found, and the role of DGCR5 in carcinogenesis remains controversial, many studies have reported DGCR5 as an oncogene or tumor suppressor 18, 20, 30. In the current study, we investigated the specific role of DGCR5 in PC with the underlying regulatory mechanism discovered for the first time. We found that DGCR5 expression was higher in PC than adjacent benign tissues and was related to the increase of histological grade, advanced TNM stage, positive lymphatic invasion and distant metastasis, and poorer patient survival. Thus, DGCR5 may be a potential diagnostic and therapeutic biomarker for PC.

We applied loss-of-function and gain-of-function approaches and demonstrated that DGCR5 performed significant effects on the biological processes of PC. Here, we validated DGCR5 was notably upregulated in PC tissues and cell lines, and DGCR5 knockdown markedly suppressed PC proliferation, DNA replication, migration, invasion, EMT process, and induced cell apoptosis and G0/G1 cell cycle arrest in vitro. Similarly, the tumor xenograft model assays displayed that DGCR5 enhanced the tumorigenicity of PC in vivo.

In general, the function of lncRNAs are mainly determined by its subcellular localization, and lncRNAs located in the cytoplasm always exert effects through the regulatory mechanism of ceRNA by sponging specific miRNAs to restore target gene expression 16, 23, 24. DGCR5 was confirmed primarily situated in cytoplasm of PC cell by FISH assays, so we supposed that DGCR5 could act as ceRNA to impact PC progression. Then we conducted RNA-seq to further identify the ceRNA network of DGCR5 in PC. Among the selected potential targets, TOP2A aroused our attention since it was the center of the PPI network and pathways analysis, in addition, TOP2A was associated with tumorigenesis and gemcitabine resistance in PC 22, 28. We observed that TOP2A expression was increased and positively associated with DGCR5 expression, but was negatively correlated with overall survival in PC. And the CCK-8 results showed that silence of TOP2A dramatically inhibited PC cell proliferation and the proliferative effects induced by DGCR5 overexpression.

To complete the ceRNA network of DGCR5, we performed bioinformatics analysis and miR-3163 was predicted to be the link between DGCR5 and TOP2A. We first examined the relation of the expression of DGCR5, miR-3163 and TOP2A, then conducted dual-luciferase assays and proved that miR-3163 could directly bind to DGCR5 and TOP2A. These results suggest the DGCR5/miR-3163/TOP2A axis as a regulatory mechanism in PC.

Wnt/β-catenin pathway has been demonstrated to play an important role in PC progression, therefore, we focused on this signaling pathway to characterize the molecular mechanisms of DGCR5 in mediating PC progression. We determined the expression of several key regulators by IHC staining and western blot, and found that DGCR5 activated the pathway, whereas TOP2A knockdown suppressed the activation induced by DGCR5. Our data suggest that DGCR5/miR-3163/TOP2A axis mediates pancreatic carcinogenesis by modulating wnt/β-catenin pathway.

Gemcitabine-based treatment is the most widely used chemotherapy for PC patients, however, chemoresistance remains a major obstacle to its clinical success 28, 31. Therefore, it is of particular importance to enhance the chemosensitivity of gemcitabine in PC cells to improve the prognosis for patients with PC. In the present study, we observed that DGCR5 knockdown increased the gemcitabine sensitivity of PC cell lines, and DGCR5 overexpression had exactly the opposite effect. To the best of our knowledge, we provide the first evidence for the function of DGCR5 on the gemcitabine sensitivity of PC, suggesting DGCR5 as a promising therapeutic target to improve the effect of chemotherapy of PC.

Recent studies manifested that the transcription factor PAX5 is highly involved the regulatory network of human malignancy as an oncogene via its transactivation function 31, 32. However, the effects of PAX5 in PC haven't yet been studied. In our study, we found that PAX5 can directly bind to the promoter region of DGCR5 and performed similar transactivation function. As revealed by these results, PAX5 acts as an upstream regulator in the ceRNA network of DGCR5. However, more in vitro and in vivo assays need to be performed to further clarify the interactions between PAX and DGCR5.

In summary, we characterized the role of DGCR5 in PC for the first time, that DGCR5 is activated by PAX5 and promotes PC progression by sponging miR-3163 to regulate TOP2A and activating wnt/β-catenin pathway (Figure 8F). Consequently, DGCR5 may be a potential diagnostic biomarker and therapeutic target for PC.

## Supplementary Material

Supplementary figure.

## Figures and Tables

**Table 1:** DGCR5 expression and clinicopathologic characteristics in pancreatic cancer (PC).

		DGCR5 expression			
Variables	Cases	Low	High	χ2 value	P value
Gender					
Male	30	14	16	0.6333	0.4261
Female	8	5	3		
*Age (years)*					
< 60	16	6	10	1.727	0.1888
≥ 60	22	13	9		
*Tumor Size (cm)*					
< 2	12	8	4	1.949	0.1627
≥ 2	26	11	15		
***Histological grade***					
High/ Moderate	18	15	3	**15.2**	<0.0001***
Low	20	4	16		
***TNM Stage***					
I-II	24	15	9	**4.071**	0.0436*
III-IV	14	4	10		
***Lymphatic invasion***					
Positive	22	6	16	**10.8**	0.001**
Negative	16	13	3		
*Vascular infiltration*					
Positive	10	4	6	0.5429	0.4613
Negative	28	15	13		
***Distant metastasis***					
Positive	5	0	5	**5.758**	0.0164*
Negative	33	19	14		

**Table 2:** Univariate and multivariate analysis of prognostic factors in pancreatic cancer patients.

		Univariate analysis		Multivariate analysis	
Variables		HR (95% CI)	P value	HR (95% CI)	P value
***DGCR5 expression***	High/Low	**3.067 (1.488-6.318)**	0.002*	**2.318 (1.074-5.000)**	0.032*
Gender	Female/Male	1.695 (0.758-3.792)	0.199	—	—
Age (years)	≥ 60/<60	1.025 (0.527-1.990)	0.943	—	—
Tumor Size (cm)	≥ 2/<2	0.908 (0.450-1.833)	0.788	—	—
Histological grade	High&Moderate/ Low	1.571 (0.810-3.047)	0.182	—	—
***TNM Stage***	III-IV/I-II	**3.324 (1.596-6.923)**	0.001*	**2.400 (1.104-5.218)**	0.027*
Lymphatic invasion	Positive/Negative	1.253 (0.644-2.439)	0.507	—	—
Vascular infiltration	Positive/Negative	1.914 (0.909-4.029)	0.087	—	—
***Distant metastasis***	Positive/Negative	**4.563 (1.645-12.658)**	0.004*	—	—

HR, hazard ratio; CI, confidence interval, —, not included. *Statistical significance, P < 0.05.

## Abbreviations

PC: pancreatic cancer
lncRNA: long noncoding RNA
DGCR5: DiGeorge syndrome critical region gene 5
TOP2A): DNA topoisomerase 2-alpha
PAX5: Paired Box 5
ceRNA: competing endogenous RNA
siRNA: small interfering RNA
NC: negative control
MOI: multiplicity of infection
qRT-PCR: quantitative real-time PCR
FISH: fluorescence in situ hybridization
IHC: immunohistochemistry
PPI: Protein-protein interaction
KEGG: Kyoto Encyclopedia of Genes and Genomes
OS: overall survival
EMT: epithelial-mesenchymal transition
DEG: differentially expressed gene
