# Supplementary material for: DGCR5 is activated by PAX5 and promotes pancreatic cancer via targeting miR-3163/TOP2A and activating Wnt/β-catenin pathway
Source: Int J Biol Sci. 2021 Jan 1;17(2):498–513. doi: 10.7150/ijbs.55636 (PMC7893588; doi:10.7150/ijbs.55636)

Figure S1. Rescue experiments using Wnt/β-catenin pathway inhibitor MASB. (A) CCK-8 assay, (B) clone formation assay and (C) transwell assay were conducted in NC or DGCR5-overexpressed PANC1 cells with 0.1% DMSO or 5μM MSAB.

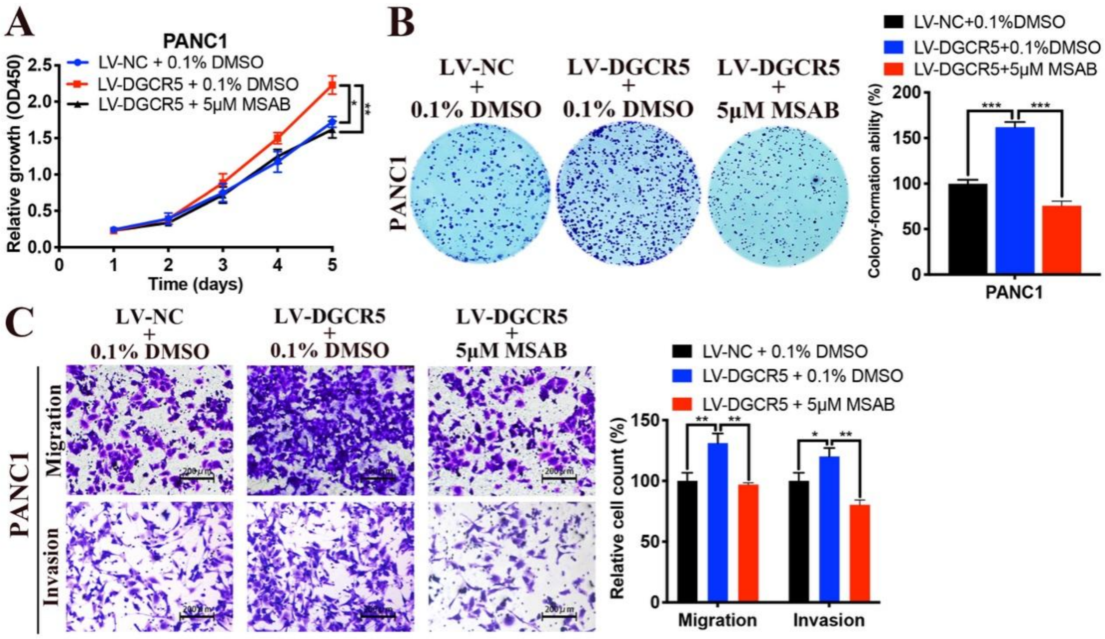

Supplement: Supplementary file 1 — Supplementary figure. [file ijbsv17p0498s1.pdf]
